# Supplementary material for: Intention to use vasectomy and its associated factors among married men in Debre Tabor Town, North West Ethiopia, 2019
Source: PLoS One. 2020 Sep 3;15(9):e0238293. doi: 10.1371/journal.pone.0238293 (PMC7470275; doi:10.1371/journal.pone.0238293)
Supplement: S1 File — (DOCX) [file pone.0238293.s001.docx]

Appendix 2 Consent form

Good morning/afternoon my name is _______________I am working as data collector in study conducted by Alemu Degu ; Midwifery Department, College of Medicine and Health Science of Debre Tabor University. The title of the study is intention to use vasectomy and its associated factors among married men in Debre tabor Northwest Ethiopia. I am interviewing clients on intention to use vasectomy and associated factors in order to collect information necessary for developing appropriate strategies to increase male involvement in FP, so that, married men like you will develop appropriate knowledge, and have intention to use vasectomy as a contraceptive method for future life. To attain this purpose, you are honestly and genuinely requesting to answer the questionnaires. You were selected to participate in this study just by chance. I expect the interview take about30-35 minutes. There can be more than one answer as given on the alternative choices or opinions. Your name or any identifying information will not be registered. You may refuse to answer any question and choose to stop the interview at any time, but the information you provide us is extremely important and valuable, as it will help the Government for formulating strategy on health sector to increase male involvement in FP. If you have any questions about the study, you can ask.

Principal investigator name: -Alemu Degu phone no: -0920770887

email: -degualemu53@gmail.com

Would you participate in our study? Yes --------No------

Signature of participant-------------------------

Date of interview: -----/---------/-----

Name of data collector-------------------

Date--------/------------/------ signature ------------------------

Questionnaire code---------

Appendix 3 English version questionnaire

**Part I: socio-demographic characteristics**

| No | Questions | Categories & responses | Skip to |
| --- | --- | --- | --- |
| 101 | Your age? | --------------------year |  |
| 102 | Ethnicity | 1.Amhara  2.Oromo  3.Tigray  4. Gurage  5. Other(specify)------------ |  |
| 103 | your religion? | 1.Orthodox  2. Protestant  3.Catholic  4. Muslim  5. Other (specify) |  |
| 104 | Your education level | 1. unable to read and write  2. Read and write  3 primary (1-8)  4 secondary (9-12)  5.college/university and above |  |
| 105 | Wife educational level | 1. unable to read and write  2. Read and write  3 primary (1-8)  4 secondary (9-12)  5.College/university and above |  |
| 106 | Your occupation | 1. Civil servant  2. Has private business  3. Employed in private sector  4.Daily laborer  5.. others (specify)------- |  |
| 107 | Wife occupation | 1.house wife  2.Civil servant  3. Has private business  4. Employed in private sector  5.others (specify)------- |  |
| **Part II: Reproductive health related variables** | | | |
| No | Questionnaire | Categories & responses | Skip to |
| 201 | How long you lived with your wife | --------------------- |  |
| 202 | Number of children alive | Male----------  Female----------- |  |
| 203 | Do you discuss about family planning methods with your partner | 1.Yes  2.No |  |
| 304 | Do you get emotional support from your partner to use FP. | 1.yes  2.no |  |
| 305 | Do you complete your family size | 1.yes  2 no |  |
| 206 | If no future desire number of children | Male----------  Female------------- |  |
| **Part III: knowledge factor questionnaires** | | | |
| No | Questionnaire | Categories and responses | Skip to |
| 301 | have you heard about male sterilization (vasectomy)? | 1.Yes  2. No |  |
| 302 | Do you know that vasectomy is permanent and irreversible | 1.Yes  2.No |  |
| 303 | Do you know that vasectomy requires minor surgical procedure? | 1.Yes  2.No |  |
| 304 | Do you know that seminal fluid during ejaculation are present after vasectomy? | 1.Yes  2.No |  |
| 305 | **Do you know how vasectomy works** | 1.Yes  2.No |  |
| 306 | Do you know that vasectomy is done in Ethiopia without any charge? | 1.Yes  2.No |  |
| 307 | Do you know where vasectomy/male sterilization service is available? | 1. Yes  2. No |  |
| 308 | have you heard that who can use vasectomy as a family planning method option? | 1.Yes  2.No |  |
| 309 | If yes for Q308 who are they | 1.Any married men of reproductive group  2.marred men who complete their family size |  |
| **Part IV Attitude related questionnaires** | | | |
| No | Question | Category and response |  |
| 401 | FP is a responsibility of women | 1.Agree  2.Neutral  3.Disagree |  |
| 402 | Do belief that vasectomy negatively affect sexual performance/desire | 1.Agree  2.Neutral  3.Disagree |  |
| 403 | Vasectomy has side effects | 1.Agree  2.Neutral  3.Disagree |  |
| 404 | Vasectomy is not acceptable in my religion | 1.Agree  2.Neutral  3.Disagree |  |
| 405 | Vasectomy is culturally unacceptable | 1.Agree  2.Nutral  3.Disagree |  |
| 406 | I am uncertain for the future pregnancy may be happen after vasectomy | 1.Agree  2.Neutral  3.Disagree |  |
| 407 | Vasectomy is similar with castration | 1.Agree  2.Neutral  3.Disagree |  |
| 408 | vasectomy can cause physical weakness, cannot do hard work | 1.Agree  2.Neutral  3.Disagree |  |
| 409 | vasectomy can ashamed the individual in the community | 1.Agree  2.Neutal  3.Disagree |  |
| **Part VI Intention questionnaires** | | | |
| No | Question | Categories and response | Skip to |
| 501 | Do you have intention to use vasectomy for future? | 1.Yes  2.No | If no skip to Q.503 |
| 502 | If yes when | 1.within 5 years  2.within 5-10 years  3.within 10-15 years  4.after 15 years |  |
| 503 | If your answer is no for Q.501 why? (more than one answer can be possible) | 1.lack of information  2.Service is not available  3.Need more children  4.Fear of procedure  5.Fear of side effect  6. its irreversibility  7.Make sexually inactive  8.partners opposition  9.Not allowed religiously  10.Lack of trained providers  11.lack of roll model  12.Other(specify)-------- |  |
