# Supplementary material for: Intention to use vasectomy and its associated factors among married men in Debre Tabor Town, North West Ethiopia, 2019
Source: PLoS One. 2020 Sep 3;15(9):e0238293. doi: 10.1371/journal.pone.0238293 (PMC7470275; doi:10.1371/journal.pone.0238293)
Supplement: Attachment — Submitted filename: point by point response (Response to Reviewers)..docx [file pone.0238293.s002.docx]

**To Plose One Academic Editor (Sphiwe Madiba, DrPH).**

**PONE-D-19-27356**

**From Alemu Degu**

**First of all I would like to say thank you very much for your timely response and for your interesting comments. And my heart full gratitude to all reviewers those giving essential comments and questions by devoting their golden time.**

**Point by point response to editors**

**I. General comments**

1. Please ensure that your manuscript meets PLOS ONE's style requirements, including those for file naming. The PLOS ONE style templates can be foundat<http://www.journals.plos.org/plosone/s/file?id=wjVg/PLOSOne_formatting_sample_main_body.pdf>andhttp://www.journals.plos.org/plosone/s/file?id=ba62/PLOSOne_formatting_sample_title_authors_affiliations.pdf

**Response 1: I accept the comment and I have fully follows the journals quid line (style requirements).**

2. Please include additional information regarding the survey or questionnaire used in the study and ensure that you have provided sufficient details that others could replicate the analyses. If you developed and/or translated a questionnaire as part of this study and it is not under a copyright more restrictive than CC-BY, please include a copy, in both the original language and English, as Supporting Information.

**Response 2: I accept the comment and I have incorporated the questionnaire as additional information.**

**II specific comments**

A. **On introduction**
1. Please revise the introduction to provide a generalized background vasectomy and its utilization, benefits, family planning, involving men in reproductive health, factors associated with low uptake of vasectomy and opinions and attitudes of men about vasectomy. There is adequate and recent data on the topic in sub Saharan Africa

**Response: I accept the comment and I have incorporated and rewrite the introduction and discussion part of the main document.**

B. **Methods**
1. Please follow the journal’s standard subheadings for the methodology.
2. Integrate the operational definitions with the measures and data analysis, write as a narrative, and remove the bolding from the words.

**Response: I accept the comment and I have followed the journals guideline in all aspects.**

C. **Data collection**
1. Provide details on the tool and type of questions that were asked. Was the tool validated, how was intention measured-the type of question, what does future intention mean- is there a time factor on the future?

**Response: I accept the comment and I have included in detail of the tool and type of questions. The tool was validated by those individuals who have experienced in related subject matter and future intention means view, thinking or expectation to use /yes there is time factor. See on the manuscript result part.**

**D. Results**

1.	Please follow the standardized categories for age and reanalyse the data accordingly, correct for all the tables and narratives in the document.

**Response: I accept the comment, but I have consider the previous related research age categories for comparison and discussion purpose and no participant age <20 years at the time of data collection, since the number of respondents are small those are aged in between 50 -59, 60-69, 70-79...merged as age >=50 years.**

D. **Discussion**
1. The discussion need a revision in line with the introduction
2. Please tell us about those who intended to use vasectomy-what were their demographics before you do the comparisons with other studies, first in Ethiopia and other countries
3. You spend too much effort of why your study findings are different from those of others and not on the implications of your findings.

**Response: I accept the comment and incorporated to the main document (manuscript) based on your comments.**

**E. Conclusion**

1. Improving level of knowledge and attitude towards vasectomy is an essential strategy-detail on how this will be done

2. The document should be copy edited for language and grammar by a professional language editor before submitting your revised manuscript.

**Response: I accept the comment and the question; we can improve the level of knowledge and attitude by giving detail awareness on the methods and benefit of it and by avoiding false remorse that talking about vasectomy. We have seen the language we ourselves the author +online grammar checker +by professional language editor.**

II. Point by point response to Reviewers

For Reviewer#1

#1: This paper addresses an important topic – vasectomy and whether men would be willing to use the method and the characteristics of men expressing such willingness. The title of the manuscript is misleading = it says “intention” but the variable in the paper is “willingness” – those are not the same thing. The authors should be clear what the men were asked out (intention or willingness – and say in the paper the question/questions asked about willingness or intention to use.

**Response#1: I accept the comment and the question widely. Yes all write, Intention and willingness is not the same thing. Where taking to Intention it is mostly explains futures expectation, thinking but the possibility may or may not. Whereas willingness is being willing to do that thing on the time or future. Generally Intention is some body’s view, thinking, expectation to the future and it is somewhat light /superficial whereas willingness is some bodies willing to act/ do or to perform and it is somewhat hard.**

**#2:** Knowledge of vasectomy: The authors say that knowledge of vasectomy was measured through 9 questions – but do not say what those questions are. The questions should be included in the paper.

Attitudes about vasectomy: It is not clear how knowledge scores can translate into positive or negative attitudes towards vasectomy. One could have good knowledge and still have a negative attitude about the method. What does “emotional support” from their partners to use vasectomy mean? How was this question asked? Were the men asked about current contraceptive use by themselves or their wives/partners?

**Response#2: I accept the comment and the question and we have included questions that we used to measure Knowledge of vasectomy in the edited manuscript. Yes write that those having good Knowledge of vasectomy may have a negative attitude about the method. And there are attitude measurement questions and knowledge measurement questions separately. ‘Emotional support’ to mean that; have you got a support or volunteer from wives, I think we use emotional inappropriate word selection and make the confusion. And we are going to asking like this “Do you get support from your partner to use FP?” Currently we accept the comment and corrected like this. Do you get support from your partner to use FP?”**

#3. Willingness (or intention?) to use: The authors should explain whey they did not ask the men if they knew where they could get a vasectomy and what would prompt them to actually have a vasectomy in the future.

Response #3: I accept the comment and the question, but the question is not clear ; in my understanding to mean that place of vasectomy ; And we are asking the availability of the service like this “Do you know where vasectomy/male sterilization service is available? So place of vasectomy is answered by this question.

#4: Furthermore, the findings of willingness or intention need to be put in the context of the availability of vasectomy in the country. Without available services, men will not be able to act on their willingness or intentions.

Response #4: I accept the comment and the availability of the service is largely there. In any district hospital, general hospital can be performed. And the service also given in the study area.

#5; The current comparison of the findings with findings from studies in other countries is interesting, but ultimately without expanded services, knowledge and willingness (or intention) is not sufficient. The authors could use the findings that are fairly consistent across studies to say that their seems to be latent interest in vasectomy that is not being satisfied with programming.

Response #4: I accept the comment and the service is at large present in our country. But insufficient number of users is there?

#5. The authors should note that the sample in this study are highly educated (63% have a college education or above, and half the wives also have a college education or above) – what is the percent of the population in Ethiopia with a college education? The authors should explain why the town of Debre Tabor was selected for the study.

**Response #5: I accept the comment and questions ; the sample in our study is highly educated which is 63% ; the reason is that the study conducted is in the town and small place and may easily accessible to education whereas the general population educational level in Ethiopia is less than this is due to the above explanation . The reason I select the study area is ; since am working there , I am not still see the procedure of vasectomy even the service is available . Due to this fact I intended to assess the intention and its factors in this area.**

#6; With that said, the men in this study could be vanguard users of vasectomy, so the findings are useful for advocating for expansion of vasectomy services in Ethiopia. It would be useful for the authors to refer to Ethiopia’s FP Costed Implementation Plan 2016-2020 to see if vasectomy is covered in it (http://www.healthpolicyplus.com/ns/pubs/2021-2030_EthiopiaCIPNov.pdf). FHI360 has prepared a brief on promoting vasectomy in Ethiopia that also might be helpful (https://www.fhi360.org/sites/default/files/media/documents/resource-vasectomy-evidence-ethiopia-final.pdf).

References should be listed by Last Name, First name (currently first names and last initial are included in the references).

This paper would benefit from a more thorough literature review on vasectomy and programming for men. Some other references to consider:

Vasectomy: A Long, Slow Haul to Successful Takeoff, James D Shelton and Roy Jacobstein, Global Health: Science and Practice December 2016, 4(4):514-517; https://doi.org/10.9745/GHSP-D-16-00355 Glob Health Sci Pract. 2016 Dec 23; 4(4): 647–660.

A Review of 10 Years of Vasectomy Programming and Research in Low-Resource Settings, Dominick Shattuck, Brian Perry, Catherine Packer, and Dawn Chin Quee , Global Health: Science and Practice, Published online 2016 Dec 23. doi: 10.9745/GHSP-D-16-00235

Hardee, K, M Croce-Galis, and J Gay. 2017. “Are Men Well Served by Family Planning Programs?” Reproductive Health. 14(14). DOI: 10.1186/s12978-017-0278-5.

Ross, J, and K Hardee. 2016. “Use of Male Methods of Contraception Worldwide.” 2016. Journal of Biosocial Science. Published online.

Response #5: I accept the comment and I have revised the literature based on your comment and suggestions.

Thank you !!!.

Alemu Degu

For Reviewer#2

Even though the topic is of public health importance as we strive to promote the use of male methods in the family planning method mix, the manuscript as it is now is very poorly written. It is extremely difficult to understand the content because of the poor quality of English. Additionally, the sampling procedure is inadequately explained - for example how the first household was selected in each kebele and how was the sample size for each kebele arrived at? It is also unclear how 'the next eligible household located in the clockwise direction was visited..' Another issue is that the authors did not provide an operational for 'intention to use vasectomy' and the Table titles are inappropriate.

The manuscript as it stands now will require major revisions, and should be seen by an English proof-reader before re-submission

**Response: I accept the comment and questions; I have taken the comment as a comment and corrected the manuscript based on your comment. Largely the issue is on grammar and language; I accept it edited at much as possible with online grammar and language checkers and with my friend fluent English speaker in my university. The first house hold is selected rondomely by lottery method for each kebele, after the number of households with married men in each kebele were found from the kebele registration book. Then the study households were selected from each kebele through simple random sampling technique by using computer generating table starting from kebele one from a random start point. One married man per household was interviewed. See it again I have operationalized it . The table title “ I have corrected based on the journal guideline. Thank you!!!.**

Alemu Degu
