# Supplementary material for: Intention to use vasectomy and its associated factors among married men in Debre Tabor Town, North West Ethiopia, 2019
Source: PLoS One. 2020 Sep 3;15(9):e0238293. doi: 10.1371/journal.pone.0238293 (PMC7470275; doi:10.1371/journal.pone.0238293)
Supplement: Attachment — Submitted filename: point by point response to reviewer.docx [file pone.0238293.s003.docx]

**To Plose One Academic Editor**

**PONE-D-19-27356R1**

**From Alemu Degu**

**First of all I would like to say thank you very much for your timely response and your actively involvement on the comments and coordination without hesitating by the current issue (COVID 19). And my heart full gratitude again reaches to all reviewers those giving a measurable, fruitful and observable comments and questions by devoting their golden time and with the challenge of COVID19. I am happy to see you the next time and year. God bless to you and us!!!**

I. Point by point response to Reviewers

For Reviewer#1

1. The authors describe intention to use vasectomy as “willingness of respondent to use vasectomy as a contraceptive method for future time.” This mixed intention and willingness, which are not the same things. Intention is stronger than willingness and implies that the man is planning to use vasectomy. Willingness implies that he might be interested in using it at some time in the future. The authors need to be clear what was asked – otherwise this seems like a vague outcome that might be more due to respondent courtesy bias than actual interest in using the method

**Response#1: We accept the comment and the question. Yes all write, Intention and willingness is not the same thing, even though some scholars give they are synonym at some point of time. As we have searched and reading different articles and different scholars point of view “Intension to use something” is the view of futurity, planning to use in the future, their future motivation to use and their effort to use in the future whereas** “**willingness** **to use something” is being willing to do that thing on the time, readiness to use and openness to perform or act. Therefore based on these and our consensus we have tried to assess their intension to use vasectomy as a contraceptive method for future time rather their** **willingness. We have tried to ask the respondents on part VI question number 501and 502”Do you have intention to use vasectomy for future? If yes when? “; on these we want to assess their future planning or view rather willingness. We can directly interpret the above question like this one “Do you have plan to use vasectomy for future? If yes when? “**

**And also we have assessed published articles related to this topic and the variables were adapted from previously published articles.**

2. Is there a reason the men were not asked if they (their wives) were currently using a contraceptive method? Most of the men said they had completed their family size, so it would be important to know if they / their wives were currently doing something to avoid pregnancy.

**Response#2: We accept the comment and the question. This is a very easy but serious issue. We are making some systematic error that makes the authors confused. Around 281 respondents had completed their family size and 116 respondents have not completed. We have tried to ask 116 respondents on future desire no of children but wrongly we report 281 respondents, we make a mistake on copy paste to the table. Those 281 respondents no need of asking about their future desire no of children, since they side already completed family size. Generally those men who have (their wives) were currently using a contraceptive method were asked about future intention to use vasectomy as contraceptive method unless their wives used permanent methods like bilateral tubal ligation.. or both those complete their family size and not complete were asked about future intention to use vasectomy as contraceptive method. We have corrected on the manuscript.**

3. Figure 2 is labeled “reason not to use vasectomy for future life” – is that how the question on intention to use was asked? This is likely a translation issue – what does it mean to use a method for future life?

**Response#3: We accept the comment and the question. Yes it makes some confusion on word utilization. And we have corrected as “Percentage distribution of study participants by reason not have intention to use vasectomy for future life ; Debre Tabor Town, North West Ethiopia, March 05-April 15 2019”.**

4. Figure 2 shows that 74% said the reason not to use vasectomy was lack of awareness – yet 46.6% said they had heard of vasectomy according to Table 3. If nearly half of the men said they had heard of vasectomy, how could three-quarters say unfamiliarity with the method was a reason not to use it?

**Response#4: We accept the comment and the question. To avoid these confusion better to describe difference between awareness and simply heard about vasectomy. Having awareness about vasectomy is that having detail knowledge of vasectomy about its purpose, procedure, side effect and advantage whereas heard about vasectomy is simply hear the presence of vasectomy not have detail knowledge. Therefore those having heard about vasectomy might or might not have awareness but those have awareness were heard about vasectomy. Therefore even though 46.6 % of respondents heard about vasectomy, they may or may not have awareness. Mostly hearing information is easy but when we asking how, for home, when…Is not answered by the respondents.**

5. The discussion would be strengthened if it focused less on how the findings of the study match other studies and more on the programmatic implications for Ethiopia – how the findings from this study can improve access to and use of vasectomy in Ethiopia.

**Response#4: We accept the comment and the question. And we have tried to edit based on the comment.**

6. The manuscript still needs English language editing.

**Response#4: We accept the comment and the question. And we have tried to edit as much as possible with online grammar and language checkers and with my friend fluent English speaker in my university.**

- **Thank you!!!**

**Alemu Degu**
