# Supplementary material for: Intention to use vasectomy and its associated factors among married men in Debre Tabor Town, North West Ethiopia, 2019
Source: PLoS One. 2020 Sep 3;15(9):e0238293. doi: 10.1371/journal.pone.0238293 (PMC7470275; doi:10.1371/journal.pone.0238293)
Supplement: Attachment — Submitted filename: point by point response to reviewer.docx [file pone.0238293.s004.docx]

**To Plose One Academic Editor**

**PONE-D-19-27356R2**
**From Fentahun Yenealem**

**First of all I would like to say thank you very much for your timely response and your actively involvement on the comments and coordination without hesitating by the current issue (COVID 19). And my heart full gratitude again reaches to all reviewers those giving a measurable, fruitful and observable comments and questions by devoting their golden time and with the challenge of COVID19.**

I. Point by point response to Reviewers

For Reviewer#1

Q1. Table 3 shows that 53.4% of the men had NOT heard about vasectomy. I should have caught this the first time I reviewed the paper. These men should have been skipped out of responding to other questions about vasectomy – if they haven’t heard of the method, how can they respond to specific questions about the method? The rest of the analysis should only be done on the 46.6% of the men who had heard of vasectomy. The conclusion that lack of knowledge about the method is an impediment to expanding use of vasectomy is still important. And then knowing what men with knowledge of the method think about it and their intention of using it will be programmatically useful.

**Response#1: We accept the comment and the question. Yes alright, we have made a great mistake on using language /taking or writing the manuscript rather data collection; we using Heard about vasectomy to mean or replaces that Do you know that vasectomy is a contraceptive method by cutting and ligating Vass deference? So this is assessing the knowledge of men’s about its procedure. Yes you are right in previous situation completely it is skip question; but wrongly usage of language. If you have time it is possible to see and check its consistency in additional file 1. We have made correction based on the comment both track change and clear manuscript.**

Q2. Also, something I noticed rereading the paper – what does it mean in Table 2 that 74% said they got emotional support from their partners to use vasectomy? Again, how could they get support from their partners that they haven’t heard of?

**Response#2: We accept the comment and the question. Yes alright, the question might be answered in the above response since heard of vasectomy is corrected accordingly.**

Q#3: The discussion would be strengthened if it focused less on how the findings of the study match other studies and more on the programmatic implications for Ethiopia – how the findings from this study can improve access to and use of vasectomy in Ethiopia.

**Response#4: We accept the comment and the question. And we have tried to edit based on the comment and based on your recommendation.**

Q#4.The manuscript still needs English language editing.

**Response#4: We accept the comment and the question. And we have tried to edit as much as possible with online grammar and language checkers and with my friend fluent English speaker in my university.**

- **Thank you!!!**

**Fentahun Yenealem**
