# Supplementary material for: Intention to use vasectomy and its associated factors among married men in Debre Tabor Town, North West Ethiopia, 2019
Source: PLoS One. 2020 Sep 3;15(9):e0238293. doi: 10.1371/journal.pone.0238293 (PMC7470275; doi:10.1371/journal.pone.0238293)
Supplement: Attachment — Submitted filename: point by point response to reviewer.docx [file pone.0238293.s005.docx]

**To Plose One Academic Editor**

**PONE-D-19-27356R3**
**From Fentahun Yenealem**

**First of all I would like to say thank you very much for your timely response and your actively involvement on the comments and coordination without hesitating by the current issue (COVID 19). And my heart full gratitude again reaches to reviewer#3 that was giving a measurable, fruitful and observable comments and questions by devoting their golden time and with the challenge of COVID19.**

I. Point by point response to Reviewer

For Reviewer#3

**General comments and recommendation: We have completely accepted the general comments and recommendations especially on the abstract section. Thank you very much for your recommendation!. Following this; We have tried to give response for each comments and questions raised by the reviewer.**

**Q #1** Please discusses in the Discussion the high educational attainment and prevalent civil servant occupations of this sample: how do these compare with national Ethiopian data (perhaps compare with EDHS data, if available), and how generalizable or not are the current data?

**Response#1: We accept the comment and the question. We have made correction based on the comment both in track change and clear manuscript. We have tried to discussing with EDHS 2016 finding the educational attainment and civil servant occupations.**

**Q #2**. Perhaps also note what percent of men have 0, 1, 2 and 3 children just to share empirical data within the category of men having 3 or fewer children.

**Response#2: We accept the comment and the question. Yes alright, the question might be answered in the above response since heard of vasectomy is corrected accordingly. The total numbers of participants those having <= 3 children is 243. The empirical data without categorized to <=3 child. 1. Those have no child 25, having one child 67, having two children 113 and having three children 38. But on the manuscript we have write by category<=3 and> 3 child.**

**Q #3**. In the Introduction when presenting data on Ethiopian family planning patterns, please also share some information about the prevalence of specific types of family planning such as condoms, , etc.

**Response#3: We accept the comment and the question. And we have tried to edit based on the comment and based on your recommendation. See in the clear manuscript and track changes.**

**Q#4**. In a related vein, in the Discussion, might another factor relevant to why men 40+ have lower vasectomy intent than men 30-39 be age- and fertility-related patterning of their wives' family planning (e.g., do older men's wives more often use tubal ligation, and does that mean that men's vasectomy decisions are somewhat contingent upon those of their wives).

**Response#4: We accept the comment and the question. And we have tried corporate based on your recommendation and question.**

**Q#5.** Please state the age range of male participants, in the Abstract and Results. I am guessing that these are men aged 20 years and older, but whether men in their teens were excluded was not noted in the Methods, nor is the upper limit of male ages specified.

**Response#5: We accept the comment and the question. And we have tried to edit based on the comment and based on your recommendation. We are not excluded those teenager participants. But on interview no participants <20 and >56 years old. That is why we are not describing in the method section. And we have made correction based on your comment on the age range in between 20-56 years. See in the clear manuscript and track changes.**

**Q#6.**  in the Methods section, please craft a clearer sentence that specifies what the cutoffs are for differentiating good and poor knowledge.

**Response#6: We accept the comment, recommendation and the question. But we have been describing it in the method section specifically in the sub heading of Measurement and data collection procedure** (line 11-13).

**Q#7:** Does the item "Get emotional support from partner" refer to emotional support generally, or specifically to family planning support?
**Response#7: We accept the comment, recommendation and the question. In this situation** get emotional support from partner" refer to emotional support specifically to family planning support.

**8#** My major concern is that the writing quality is inadequate for the journal, and that a more professional editorial process is necessary before a final version can be accepted.

**Response#8. We accept the comment and the question. And we have tried to edit as much as possible with online grammar and language checkers and with my friend fluent English speaker in my university.**

- **Thank you!!!**

**Fentahun Yenealem**
