# Supplementary material for: Intention to use vasectomy and its associated factors among married men in Debre Tabor Town, North West Ethiopia, 2019
Source: PLoS One. 2020 Sep 3;15(9):e0238293. doi: 10.1371/journal.pone.0238293 (PMC7470275; doi:10.1371/journal.pone.0238293)
Supplement: Attachment — Submitted filename: Point by point response 4.docx [file pone.0238293.s006.docx]

**To Plose One Academic Editor**

**PONE-D-19-27356R4**
**From Fentahun Yenealem**

**First of all I would like to say thank you very much for your timely response and your actively involvement on the comments and coordination without hesitating by the current issue (COVID 19). And my heart full gratitude again reaches to reviewer#3 that was giving a measurable, fruitful and observable comments and questions by devoting their golden time and with the challenge of COVID19.**

I. Point by point response to Editor and Reviewer

Q# 1. Reviewer #3: The authors have addressed adequately the few substantive questions/comments I raised in the last round of review. I do not request any additional substantive edits. However, I still feel that the manuscript needs copyediting before it could be published. I do not know if PLoS ONE provides that service. If not, then it is worth noting that some formatting issues and grammatically awkward phrases remain in this revision. I don't believe this is the task of a reviewer to undertake all of these, as I illustrated previously with edits to the Abstract. I thus would ask that some formal copyediting process beyond what the authors have done be undertaken to ensure a final polished version of the manuscript.

**Response 1: We accept the comment and the question. And we have tried to copy edit as much as possible with online grammar and language checkers and with my friend fluent English speaker in my university.**

- **Thank you!!!**

**Fentahun Yenealem**
